# Supplementary material for: Flexible joints performance assessment of additive manufacturing FDM 3D printed TPU
Source: PLoS One. 2025 Nov 13;20(11):e0336401. doi: 10.1371/journal.pone.0336401 (PMC12614568; doi:10.1371/journal.pone.0336401)
Supplement: Attachment — Submitted filename: Response to Reviewers.pdf [file pone.0336401.s002.pdf]

Original Manuscript ID: PONE-D-25-26891
Original Article Title: “Flexible joints performance assessment of additive manufacturing FDM 3D printed TPU.”

To : Ph.D Himadri Majumder, *PLOS ONE Academic Editor*
Re : Response to reviewers

Dear Editor,

Thank you for allowing a resubmission of our manuscript, with an opportunity to address the reviewers' comments.
We are uploading (a) A rebuttal letter that responds to each point raised by the academic editor and reviewers, labeled 'Response to Reviewers'. (b) A marked-up copy of the manuscript that highlights changes made to the original version, labelled 'Revised Manuscript with Track Changes'. (c) An unmarked version of the revised paper without tracked changes labelled 'Manuscript'.

We trust that the revised manuscript now meets the reviewers' expectations. We again thank the reviewers for their valuable insights, which significantly improved the quality of this work.

Best Regards,

Daniel Rodríguez Flores, et al.

1 Response to reviewers:

The authors appreciate all reviewers' comments and suggestions and are confident that the corrections outlined in this document fulfill their expectations.

It is worth mentioning that cross-references to bibliography and figures refer to this document (Response to Reviewers) and only when specified, refer to those of the newly submitted manuscript.

1.1 Reviewer # 1: Concern # 1 Abstract Clarity

The abstract provides a general overview but lacks clarity in explaining the novelty of the work. Consider explicitly stating what makes this study unique compared to existing literature on flexible joints or lattice-based sandwich structures.

Author response:

We appreciate the reviewer's comment. The readability and structure of the abstract are improved following the guide below: (i) contextualization (positioning of the scenario to be evaluated), (ii) formulation of the research question, (iii) methodology, (iv) presentation of results, and (v) conclusion (scientific contribution).

Compared to existing literature, where the characterization of the 3D-printed material is focused on behaviours consequence of the 3D-printing process and possible non-linear behaviours. This study aims to research the effects of 3D-printed parameters and geometric dimensions, i.e, printing density, layer thickness, raster angle, length, width, and height on the stiffness/spring and damping coefficient. The Box-Benken design of experiments is conducted to obtain 44 different parametric combinations to characterize the damping and spring coefficients C_F, K_F, C_τ, K_τ under a dynamic load.

Author action:

As suggested by multiple reviewers, the abstract and conclusions were rewritten to improve clarity, remove statistical error values, and highlight novelty, research gaps, and applications. Specific changes include: Statistical error values have been removed from the abstract and replaced with a concise summary of the model's accuracy. The novelty of the work is explicitly stated.

1.2 Reviewer # 1, Concern # 2 Introduction Depth

The introduction mentions several prior works but could benefit from a more detailed gap analysis. Please elaborate on what specific problem remains unsolved and how this study addresses that gap.

Author response:

We agree with the reviewer's assessment. The selected application for this study is flexible joints for robots. For robotic applications, a more complex load is of interest where the application is not monotonic, unlike most loads in the reviewed research.

Compared to existing literature, where the characterization of the 3D-printed material is focused on behaviours consequence of the 3D-printing process and possible non-linear behaviours. This study aims to research the effects of 3D-printed parameters and geometric dimensions, i.e, printing density, layer thickness, raster angle, length, width, and height on the stiffness/spring and damping coefficient. The Box-Benken design of experiments is conducted to obtain 44 different parametric combinations to characterize the damping and spring coefficients C_F, K_F, C_τ, K_τ under a dynamic load.

Author action:

Pros and cons of the material extrusion process were expanded upon in the 3rd paragraph of the introduction specifically: "However, the layer-by-layer nature of the FDM manufacturing process results in limited surface and mechanical characteristics that can lead to undesired porosity, delamination, stress concentrators, reduced mechanical performance, or premature failure. The combination of printing parameters modifies the performance of the flexible joints [10], [11]".

The applications of this study were expanded on the 5th paragraph as follows: "The selected application for this study is 3D-printed flexible joints for robots. Some examples of flexible joints in robotics are for Quadruped Robots to enhance its impact buffering capability as well as reducing energy consumption [15], robot arms that mimic the free movement of the human arm with guarantee transient performance [16], miniaturized soft robots for in vitro or in vivo applications [17], Soft artificial muscles for lifting objects mimicking chameleon tongues [18] and exoskeletons for motion assistance in rehabilitation [19].".

The structure of the introduction was modified to avoid abrupt transitions and improve readability. The objective and specific research gap is defined explicitly in a new tenth paragraph. Related references "4D Printing of Composite Thermoplastic Elastomers for Super-Stretchable Soft Artificial Muscles.", "Effects of 3D-printing processing parameters on FFF parts' porosity: Outlook and trends." were added,

corresponding to references [10] and [14], and commentary on related research was expanded.

1.3 Reviewer # 1, Concern # 3 Figure Quality and Captions

Some figures (e.g., Figures 2, 4, and 8) have relatively low resolution and unclear captions. Improve image quality and ensure all abbreviations or variables are clearly defined in the captions.

Author response:

This is a great observation by the reviewer. The original DPIs of the images do not comply with PLOSONE standards. The original DPI of images 2, 4, and 8 are 97, 300, and 300 respectively.

Author action:

The original DPI of images 2, 4, and 8 increased from 97, 300, and 300 to 600. Images were modified so that the X, Y, and Z axes are on caps and match the rest of the document. Captions of images 2 and 4 were modified to ensure all variables are identified and defined.

1.4 Reviewer # 1, Concern # 4 Methodological Details

The authors should provide more detailed information about the material properties used in the simulations, including elastic modulus, Poisson's ratio, and any assumptions made during modeling. Were the lattice structures modeled as homogeneous materials?.

Author response:

This is an excellent observation raised by the reviewer. The material properties of the thermoplastic polyurethane for the flexible joints are Young modulus (E) 1.69×10^3 MPa. Poisson's ratio is 0.33, but said parameters are not used to calculate the values in the matrices $\mathbf{M}$, $\mathbf{C_F}$, $\mathbf{K_F}$, $\mathbf{I}$, $\mathbf{C}_\tau$, $\mathbf{K}_\tau$; the values are calculated using the closed form solution of the least squared problem.

$$\hat{\beta} = \left(\mathbf{X^T X}\right)^{-1} \mathbf{X^T y} \tag{1}$$

where $\mathbf{X}$ is the input variables r, $\dot{r}$, $\ddot{r}$, Θ, ω, $\dot{\omega}$, $\mathbf{y}$ are the output variables F, τ and $\hat{\beta}$ are the estimated coefficients $\mathbf{M}$, $\mathbf{C_F}$, $\mathbf{K_F}$, $\mathbf{I}$, $\mathbf{C}_\tau$, $\mathbf{K}_\tau$. In the case of the proposed experiment, the inputs are the positions, and the outputs are the forces and torques. All the values used are measured according to the design of the experiment proposed in Section 1.2. The proposed model for the flexible joint is given by Eqs (2) and (3):

$$\mathbf{M}\ddot{r}(t) + \mathbf{C_F}\dot{r}(t) + \mathbf{K_F}r(t) = F(t) \tag{2}$$

$$\mathbf{I}\dot{\omega}(t) + \mathbf{C}_\tau\omega(t) + \mathbf{K}_\tau\Theta(t) = \tau(t) \tag{3}$$

Meaning the lattice structures are not modeled, and the flexible joint is considered homogeneous, and its behavior can be represented by equations (2) and (3).

Author action:

The model section was separated from the experimental design section. A new model section 1 titled "Proposed mass-spring-damper model" was added where the equations (2) and (3) were moved to, and equation (1) was added. A new paragraph was added providing a more explicit explanation of the model's input and outputs and how to identify its parameters as follows: "where $\mathbf{X} \in \mathbb{R}^{3\times n}$ is the input variables, n is the number of data points in each experiment, $\mathbf{y} \in \mathbb{R}^{3\times n}$ are the output variables and $\hat{\beta}$ are the estimated coefficients. In the case of the proposed experiment the inputs are the positions (r, θ), speed ($\dot{r}$, ω), and velocities ($\ddot{r}$, $\dot{\omega}$), and the outputs are the forces and torques (F, τ).".

1.5 Reviewer # 1, Concern # 5 Validation of FE Model

While the paper includes numerical results, experimental validation of the finite element (FE) models is lacking. Even a brief comparison to literature data or a previous study would strengthen the reliability of the simulations.

Author response:

We appreciate the reviewer's comment. No simulations were realized in the article. All the numerical results presented are based on real data obtained from the experiment proposed in Section 1.2. The experimental setup is shown in the Fig 1. Forty-four datasets were obtained via experimentation and used to obtain the coefficients of the proposed mass-damper-spring model. A video example of the experiment is shown in: `https://youtu.be/PuYnzHvUt_I`

The proposed model is validated using a static test based on Hookes'law for torsional elements. To the best of the author's knowledge, the closest research for flexible 3D-printed materials is [1], [2], and [3], where the specimens are subjected to a single type of load, namely torsional, tension, or deflection. The application of the forces is either constant or monotonic. To expand on the case study for the selected application, a combination of loads was used.

The proposed model is a mass-spring-damper model. The proposed mass-spring-damper model is an

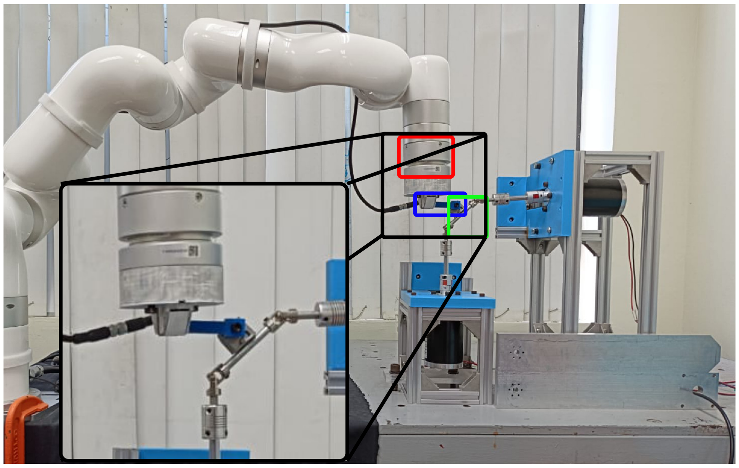

Figure 1: Experimental setup.

extension of a popular model used in the design of compliant mechanisms known as the pseudo-rigid-body model.

The FE model was considered out of the scope of the article but both the Modal and harmonic response simulations were done using an FE model. Using ANSYS 2025, a custom TPU material was defined. Using a random specimen CAD model with dimensions $l = 18mm$, $h = 14mm$, and $b = 4mm$, a mesh is defined with 3989 nodes and 2272 elements. Applying fixed supports on one side and frictionless supports on the other. The resulting vibration modes are shown in Table 1, and a screenshot of the first mode of vibration is shown in Figure 2.

Table 1: **Modes of vibration.**

**Vibrational Modes**	**Frequency**
1	3.1545
2	14.018
3	15.061
4	17.546
5	22.537
6	33.345

The resulting vibration modes are above the experimental applied frequencies, meaning the experimental behaviour does not reach the behaviour of the modes of vibration. In the case of the harmonic analysis, applying a similar force, the resulting displacements are similar to the experimental displacements.

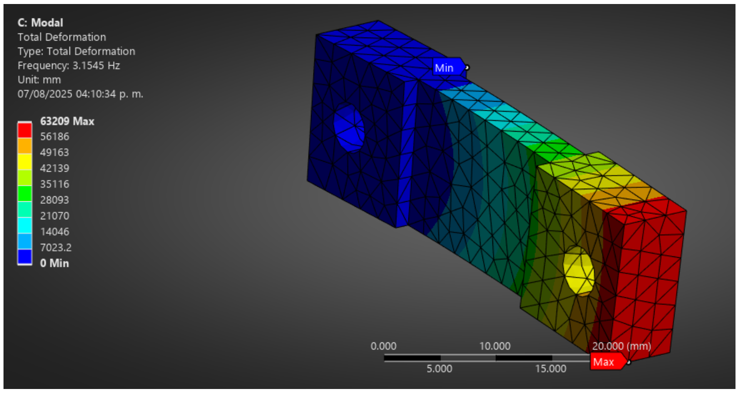

Figure 2: ANSYS modal analysis.

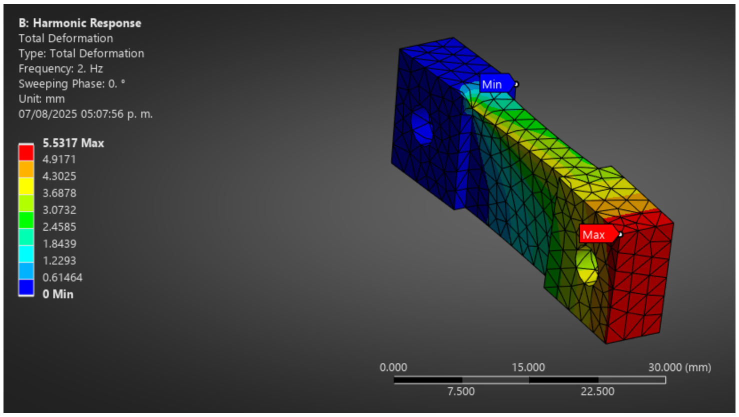

Figure 3: ANSYS harmonic analysis.

1.6 Reviewer # 1, Concern # 6 Discussion on Limitations

The paper would benefit from a section discussing the limitations of the current approach both in terms of simulation assumptions and possible real-world manufacturing constraints.

Author response:

Acknowledgement is due for the reviewer's insight. The proposed mass-spring-damper model assumes the material is homogeneous, the internal lattice structures are not modelled. Its assumed that the damping-spring coefficients obtained describe the accurate behaviour on a small region of deformations in a steady state of motion. The region of deformations is given by the sinusoidal signal amplitude (1 rad).

The results show that the 3D-printing technology can be used in the design of robots with flexible joints. Some examples include:

- Soft robots that mimic the free movement of tails and tentacles.
- Exoskeletons for motion assistance.
- Humanoids and Quadrupeds.

The manufacturing constraints can be divided into two sets: mechanical, which include the material uniformity, stress concentrators, and aesthetics, like the resolution, and unexpected porosity in the final product.

Author action:

The conclusions were rewritten to avoid repeating the results section and to communicate this research's limits and prospects, as well as possible applications in the industry and real-world manufacturing constraints as follows "The MANOVA showed the accuracy of the model and the importance of each parameter. The P-values were below 0.05 for the layer, width, and height. Among the examined parameters, the geometry parameters have the most significant roles in modifying the damping and spring coefficients, with the height being the most significant and the length the least important. Tuning the structural parameters of the 3D-printing process plays a significant role in the behaviour of the 3D-printed parts. The resulting low-damping factors show that the technology can be used in the design of robots with flexible joints to avoid the use of ball bearings. Some examples include: Soft robots that mimic the free movement of tails and tentacles, exoskeletons for motion assistance, humanoids, and quadrupeds.".

1.7 Reviewer # 1, Concern # 7 Clarity in Results Section

The results section discusses stress distribution and stiffness but should also discuss possible implications on design, such as trade-offs between flexibility and strength, or how joint orientation might impact fatigue life.

Author response:

The printing density of the specimen has the most influence on the damping but almost no influence on the spring. If a damping phenomena is to be introduced in the flexible joint it is recommended that the joint is manufactured at 100 % density. The influence of the geometric variables is associated with the capacity of the test specimen to bend. In Fig 4 the correlation heatmap is shown. The variables with the most correlation be it positive or negative with the spring, and damping coefficients are the width and height.

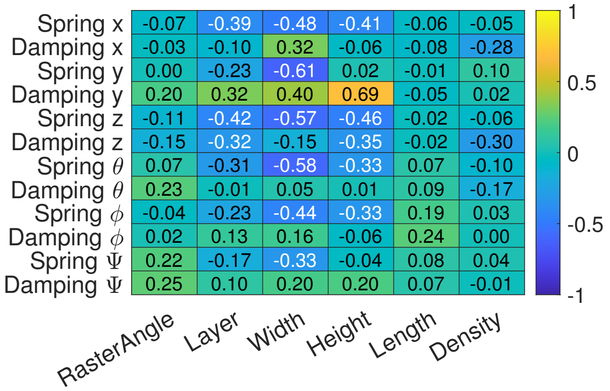

Figure 4: **Correlation Heat-map between input and output variables.**

According to the results its expected that the greater the deflection the higher the fatigue experienced. The fatigue analysis according to each D-printing parameter is considered future work.

Author action:

Discussion of the resulting coefficients and possible design applications was added to the second paragraph of section 3 Results and discussion: “The resulting spring and damping coefficients show that this type of flexible joint can be used in applications that require energy-saving and release mechanisms. The damping coefficients allow for substitution of ball bearing joints for flexible joints at the expense of reduced angular motion.”. Results were linked to design trade-offs (density vs damping, geometry vs stiffness). A note on fatigue analysis as is considered future work was added to the 3rd paragraph of the Conclusions “Future work is proposed related to the trade-offs between strength and flexibility, how joint

orientation might impact fatigue life, and the influence of lattice-based sandwich structures.”.

1.8 Reviewer # 1, Concern # 8 Grammar and Language

The manuscript requires minor grammatical corrections for fluency and readability. For example, replace “this paper investigates the joint flexibility...” with “This paper investigates the flexibility of joints...”

Author response:

This observation raised has contributed to improving the clarity of the manuscript; the document has been reviewed to correct syntax, grammatical errors, and improve readability.

Author action:

Abstract was rewritten “Given the increasing adoption of flexible 3D-printed joints in the field of robotics, it is essential to characterize the stiffness/spring coefficient and damping of printed specimens to understand the effects of various processing parameters and their interactions under different loading conditions. This study aims to research the effects of 3D-printed parameters and geometric dimensions, i.e, printing density, layer thickness, raster angle, length, width, and height. The Box-Benken design of experiments is conducted to obtain 44 different parametric combinations to characterize the damping and spring coefficients $\mathbf{C_F}$, $\mathbf{K_F}$, $\mathbf{C_\tau}$, $\mathbf{K_\tau}$ under a dynamic load.

The damping and spring coefficients are characterized using the minimum squared method. Depending on the force decomposition, the damping and spring coefficients are different in each direction. To analyse the experimental results, a MANOVA, ANOVA analysis, and a correlation heatmap are used to show that the density, layer thickness, and width have the most influence on the damping. In contrast, raster angle has the most influence on the spring coefficient.

Finally, the results show that the geometric and 3D-printing parameters play a significant role in the mechanical behaviour of flexible joints. The technology can be used in the design of robots that require energy-saving and releasing mechanisms, and to avoid the use of ball bearings under a dynamic load.”. The manuscript was modified to comply with PLOSOne format guidelines.

1.9 Reviewer # 1, Concern # 9 Conclusions Enhancement

The conclusions currently restate results without highlighting the significance. Consider briefly noting how these findings can influence future additive manufacturing design of lattice-based sandwich structures.

Author response:

Indeed the point raised by the reviewer is taken into consideration. The results show that different coefficients are obtained depending on the measure axis or angle. Increasing the printing density has the most influence on the damping instead of the spring coefficients. The raster angle has the most influence on the spring coefficients, being the highest at 0°. A finer layer thickness increases both the damping and spring effect. The width has an inverse correlation effect between its effect on damping and spring coefficients, where the correlation is positive on damping and negative on spring coefficients. The Density has the highest influence on the damping but almost no influence on the spring.

The MANOVA showed the accuracy of the model and the importance of each parameter. The P-values were below 0.05 for the layer, width, and height. Among the examined parameters, the geometry parameters have the most significant roles in modifying the damping and spring coefficients, with the height being the most significant and the length the least important.

Tuning the structural parameters of the 3D-printing process plays a significant role in the behaviour of the 3D-printed parts. The resulting low-damping factors show that the D-printing technology can be used in the design of robots with flexible joints to avoid the use of ball bearings. Some examples include: Soft robots that mimic the free movement of tails and tentacles, exoskeletons for motion assistance, humanoids, and quadrupeds. Future work is proposed related to the trade-offs between strength and flexibility, how joint orientation might impact fatigue life, and the influence of lattice-based sandwich structures.

Author action:

The conclusions were rewritten and a new paragraph was added to expand on the future work and influence on additive manufacturing as follows: “Future work is proposed related to the trade-offs between strength and flexibility, how joint orientation might impact fatigue life, and the influence of lattice-based sandwich structures.”.

1.10 Reviewer # 1, Concern # 10 References

Ensure that all references are formatted consistently and up to the journal's guidelines. Double-check citations for completeness.

Author response:

We appreciate the reviewer's concern.

Author action:

All references were checked for completeness and journal formatting using PLOSOne plos2015.bst template.

1.11 Reviewer # 2, Concern # 1

The title and keywords are appropriate.

Author response:

We appreciate the reviewer's comment.

1.12 Reviewer # 2, Concern # 2

Please improve the abstract: following the guide below: (i) contextualization (positioning of the scenario to be evaluated), (ii) formulation of the research question, (iii) methodology, (iv) presentation of results, and (v) conclusion (scientific contribution).

Author response:

We are thankful for the reviewer's detailed evaluation. The readability and structure of the abstract was improved following the guide: (i) contextualization (positioning of the scenario to be evaluated), (ii) formulation of the research question, (iii) methodology, (iv) presentation of results, and (v) conclusion (scientific contribution).

The novelty of this work focuses on the characterization of the 3D-printed material is focused on behaviours consequence of the 3D-printing process and possible non-linear behaviours. This study aims to research the effects of 3D-printed parameters and geometric dimensions, i.e, printing density, layer thickness, raster angle, length, width, and height on the stiffness/spring and damping coefficient. The Box-Benken design of experiments is conducted to obtain 44 different parametric combinations to characterize the damping and spring coefficients $\mathbf{C_F}$, $\mathbf{K_F}$, $\mathbf{C_\tau}$, $\mathbf{K_\tau}$ under a dynamic load.

Author action:

Abstract was rewritten adding a concise summary of the model's accuracy: "To analyse the experimental results, a MANOVA, ANOVA analysis, and a correlation heatmap are used to show that the density, layer thickness, and width have the most influence on the damping. In contrast, raster angle has the most influence on the spring coefficient.". Statistical error values have been removed from the abstract.

1.13 Reviewer # 2, Concern # 3

Intro. 3rd para. Please refer to the pros and cons of the material extrusion process performance (bonding, porosity, surface roughness, hardness, compression, etc.) and better define the objectives of this research. See the following very related studies to understand what I mean.
https://doi.org/10.1007/s00170-024-14232-0 (roughness)
https://doi.org/10.1080/10426914.2024.2304843 (porosity challenges)
https://doi.org/10.1007/s00170-025-15124-7 (bonding challenges)
https://doi.org/10.1080/10426914.2023.2290258 (strength)

Author response:

We are grateful for the reviewer's valuable suggestion. The layer-by-layer nature of the FDM manufacturing process results in limited surface and mechanical characteristics that can lead to undesired porosity, delamination, stress concentrators, reduced mechanical performance, or premature failure. The combination of printing parameters modifies the performance of the flexible joints [4], [5].

Author action:

Pros and cons of the material extrusion process were added to the 3rd paragraph of the introduction. The structure of the introduction was modified to add related references [4], [5] that correspond to references [10], and [14] on the main document, expand the description of related articles, and improve readability.

1.14 Reviewer # 2, Concern # 4

Intro. Then, please communicate the research gap more effectively and what new insights this research presents.

Author response:

We appreciate the reviewer's assessment. The selected application for this study is flexible joints for robots, where characterizing their behavior under torsional and flexural loads is the main objective. It is essential to characterize the mechanical performance of printed specimens under various loadings.

Several studies have analyzed the mechanical response of flexible 3D-printed polymers, providing insights into their performance under various loading conditions. For instance, Sadaghian et al. [1] investigated the behavior of 15 different 3D-printed materials under monotonic torsion, observing that failure in most specimens except for TPU was brittle and sudden, initiating between layers rather than within them (i.e., filament fracture). Other types of studied loads include compression [2], tension [3], and deflection.

For robotic applications, a more complex load is of interest where the application is not monotonic, unlike most loads in the reviewed research. To adjust to the standard dynamic loadings used in robotics, a combination of loads applied at different frequencies is of interest.

Author action:

The objective and specific research gap is defined explicitly in a new tenth paragraph "Compared to past research where the characterization of the material is under a monotonic or continuous load. The main objective is characterize the stiffness/spring coefficient and damping of printed specimens under a dynamic load, followed by researching the effects of 3D-printed parameters and geometric dimensions in the mechanical characteristics of flexible joints. A combination of torsional and flexural loads are applied to FDM TPU parts, no other studies using flexural loads were found. To obtain the stiffness/spring and damping coefficients six process parameters in the manufacturing process are considered.".

1.15 Reviewer # 2, Concern # 5

Section 1.1. Please provide a clearer explanation of the selection of the tested parameter and its levels. Why does raster angle have two levels?

Author response:

The 3D-printing variable parameters were chosen assuming their maximum impact given similar research on tension and compression conditions [6, 7, 8].

The raster angle was chosen as 0 and 45 degrees, and 90 degrees was omitted, given that previous research [4] found it behaved similarly to the 0-degree condition.

For the rest of the parameters: printing density, layer thickness, length, width, and height, only the extremes were chosen, meaning levels 1 and 3. Level 2 was obtained using the Box-Behnken method to design the experiment. The levels are shown in Figure 2. The software used was Minitab 22.

Table 2: Parameters and their levels

Parameters	Levels		
	1	2	3
Printing density (%)	20	60	100
Length l (mm)	18	24	30
Width b (mm)	2	4.5	7
Height h (mm)	4	9	14
Layer thickness (mm)	0.12	0.16	0.2
Raster angle (°)	0	45	–

Author action:

In section 2.2 Design of experiments, paragraph 4, the DOE explanation about how the variable parameter levels were expanded as follows: "The experiment is designed using the Box-Behnken method in the software Minitab 22, where Table 2 presents the variables of the current research and their intervals. Levels 1 and 3 are chosen by design, and level 2 is generated by the DOE Minitab 22 tool. The least squares technique is used to fit the dynamic mass-spring-damper model shown in Eqs [1] and [2] for the linear deformations and Eqs [3] and [4] for angular deformations. This model assumes that mass m, spring coefficient c, and damping coefficient k differ depending on the axis. These conditions are given by the geometry of the test specimen, whose cross section is not symmetric, the anisotropic properties of the

material and manufacturing method, the natural frequencies, and the settling time that differ depending on the direction [47].".

1.16 Reviewer # 2, Concern # 6

Table 3. Please give the details of the experimental design. Factors, center point, etc. Give a reference that uses the same BBD experimental array, or explain where to find or how construct this experimental array.

Author response:

The number of factors is six and corresponds to the number of parameters shown in Table 3.

Table 3: Parameters and their levels

Parameters	Levels		
	1	2	3
Printing density (%)	20	60	100
Length l (mm)	18	24	30
Width b (mm)	2	4.5	7
Height h (mm)	4	9	14
Layer thickness (mm)	0.12	0.16	0.2
Raster angle (°)	0	45	–

There are eighteen center points of the variables that were determined by the DOE Minitab 22 tool and one block. The result is a total of 162 runs. The complete DOE Minitab 22 document was uploaded to https://simtk.org/projects/flex-tpu.

Author action:

In section 2.2, paragraph 4, the DOE explanation was expanded to add details of the experimental design.

1.17 Reviewer # 2, Concern # 7

Section 2.2 should be better communicated.

Author response:

We recognize the reviewer's thoughtful input.

Author action:

Section 2.2 is now Section 3.2 Model validation. The original section was rewritten as follows:

"To validate the coefficients resulting from the data-based model, a Hookes-Law-based test is proposed. One end of the test specimen is fixed to a rigid support, and at the other end, a fixed load is applied as shown in Fig [14].

To measure the position and angle, an Intel RealSense D455 binocular camera and a marker at the load end are used. A 280 g load is applied to the test specimens. The load is positioned at the length of the specimen and maintained for 600 s. The deformation of the free end of the test specimen is measured, and the angle θ and the spring constant k_θ are calculated. The moment τ_θ is also calculated based on the applied force and length of the test specimen. The resulting spring constant k_θ is then compared to the least squares numeric result.

The error between the expected spring coefficient and the resulting spring coefficient is obtained for each of the test specimens, the mean error of the 44 spring coefficients is 0.008 N/mm or 1.164% for the linear deformation x, 0.0392 N/mm or 7.09% for the linear deformation z, 0.0012 N/mm or 4.083% for the angular deformation θ, 5.5205×10^{-4} N/mm or 4.6004% for the angular deformation Ψ.".

1.18 Reviewer # 2, Concern # 8

Explain Table 4. What does the contribution in the last column mean?

Author response:

The first column "Source" is the corresponding factors and the interception of them. The second column "Statistic" corresponds to the statistical tools used, Pillai, Wilks, Hotelling, and Roy (P, W, H, R). The third column "F", corresponds to the statistical value associated with the multivariate hypothesis test, which compares the variance between groups versus the variance within groups. Column four "RSS", is the residual sum of squares used by the statistical tools. Columns five and six "DF1 and DF2", correspond to the number of degrees of freedom in the numerator and denominator, respectively. The

seventh column is the P-value. The eighth column is the "Contribution", which is a measure of how much each independent variable (factor) contributes to the multivariate separation between groups. Represents the relative importance of the effect in the total multivariate variability.

Author action:

In section 3.1, in paragraph 2 a Table 4 description of the least common columns was added "The results of the MANOVA for the x associated coefficients are displayed in Table 4. The **Statistic** column corresponds to the statistical tools used, Pillai, Wilks, Hotelling, and Roy (P, W, H, R). Columns five and six **DF1** and **DF2**, correspond to the number of degrees of freedom in the numerator and denominator, respectively. The eighth column is the **Contribution**, measures of how much each independent variable contributes to the multivariate separation between groups.".

1.19 Reviewer # 2, Concern # 9

9. Conclusions should not only repeat the discussion section but also communicate this research's limits and prospects, as well as possible applications in the industry.

Author response:

We share the reviewer's perspective on this point. Tuning the structural parameters of the 3D-printing process plays a significant role in the behaviour of the 3D-printed parts. The resulting low-damping factors show that the technology can be used in the design of robots with flexible joints to avoid the use of ball bearings. Some examples include: Soft robots that mimic the free movement of tails and tentacles, exoskeletons for motion assistance, humanoids, and quadrupeds.

Author action:

The conclusions were rewritten to expand on the influence of the research in additive manufacturing and possible applications a new 3rd paragraph was added: "Tuning the structural parameters of the 3D-printing process plays a significant role in the behaviour of the 3D-printed parts. The resulting low-damping factors show that the 3D-printing technology can be used in the design of robots with flexible joints to avoid the use of ball bearings. Some examples include: Soft robots that mimic the free movement of tails and tentacles, exoskeletons for motion assistance, humanoids, and quadrupeds. Future work is proposed related to the trade-offs between strength and flexibility, how joint orientation might impact fatigue life, and the influence of lattice-based sandwich structures.".

1.20 Reviewer # 2, Concern # 10

Please proofread the document carefully for units, typos, syntax, figures, details, legends, and text format.

Author response:

We agree with the reviewer's observation. The document has been reviewed to correct syntax and grammatical errors.

Author action:

The manuscript was proofread; units, syntax, figure legends, and tet format was corrected some specific examples include: captions on Figs 2 and 4 were changed to ensure all variables are identified. "Fig." was changed to "Fig" and "Figs", "Eq." was changed to "Eq" an "Eqs". Syntax in the introduction and Abstract was changed.

1.21 Reviewer # 3, Concern # 1

1. In the Abstract:

- **Some sentences particularly the second and third are excessively long and include multiple concepts within a single complex structure, which makes comprehension and focus difficult.**

- **Including exact statistical error values at the end of the abstract is not particularly helpful for the general reader. It is preferable to present only a concise summary of the model's accuracy.**

- **The final sentence begins with a lowercase letter ("the mean error..."), which is a grammatical error in standard English writing and should be corrected.**

Author response:

We agree with the reviewer's assessment. The readability and structure of the abstract are improved following the guide below: (i) contextualization (positioning of the scenario to be evaluated), (ii) formulation of the research question, (iii) methodology, (iv) presentation of results, and (v) conclusion (scientific contribution).

Author action:

As suggested by multiple reviewers, the abstract and conclusions were rewritten to improve clarity, remove statistical error values, and highlight novelty, research gaps, and applications. Specific changes include:

- The second sentence was removed and the third sentence modified.
- Statistical error values have been removed from the abstract and replaced with a concise summary of the model's accuracy.
- The final sentence was changed and grammar was checked.

The new abstracts' first paragraph is written as follows "Given the increasing adoption of flexible 3D-printed joints in the field of robotics, it is essential to characterize the stiffness/spring coefficient and damping of printed specimens to understand the effects of various processing parameters and their interactions under different loading conditions. This study aims to research the effects of 3D-printed parameters and geometric dimensions, i.e, printing density, layer thickness, raster angle, length, width, and height. The Box-Benken design of experiments is conducted to obtain 44 different parametric combinations to characterize the damping and spring coefficients $\mathbf{C_F}$, $\mathbf{K_F}$, $\mathbf{C_\tau}$, $\mathbf{K_\tau}$ under a dynamic load.".

1.22 Reviewer # 3, Concern # 2

In the Introduction:

- **While many references are cited, there is minimal critical analysis or synthesis of previous work. The introduction should also provide an evaluative and analytical perspective. A dedicated paragraph discussing the literature review and analyzing similar studies is recommended.**
- **The content lacks structural organization. There are abrupt transitions between general descriptions and technical details, which affect the overall coherence.**
- **Before stating the objective of the paper, it is important to clearly identify the gaps or limitations in previous research and explain how this study aims to address them.**

- **It is recommended to use the following papers. 4D Printing of Composite Thermoplastic Elastomers for Super-Stretchable Soft Artificial Muscles. 3D printed elastomers with superior stretchability and mechanical integrity by parametric optimization of extrusion process using Taguchi Method. 3D printing super stretchable propylene-based elastomer.**

Author response:

We acknowledge and support the reviewer's assessment.

Author action:

- Critical analysis of the cited references was expanded.
- The structure of the introduction was modified to avoid abrupt transitions and improve readability.
- Paragraphs 4 and 5 of the introduction were rewritten to identify the research gaps. A new 6th paragraph was added to clearly state the objective of the research.
- Added related references "4D Printing of Composite Thermoplastic Elastomers for Super-Stretchable Soft Artificial Muscles.", "Effects of 3D-printing processing parameters on FFF parts' porosity: Outlook and trends." were added, corresponding to references [10] and [14], on the 5th paragraph of the introduction, and expanded the description of related articles.

1.23 Reviewer # 3, Concern # 3

In the Materials and Methods section:

- **Although it is stated that the parameters were adopted from similar studies, the technical details or rationale behind the selection of specific values (e.g., why 0.12 or 0.16 mm for layer thickness?) are not explained. It is recommended to discuss the reasoning behind the choice of these parameters.**
- **The images showing the printing process and final printed samples are appropriate, and the schematic diagram is helpful. However, to complete this section, it is also recommended to include images of the raw materials used.**

Author response:

Indeed we agree with the reviewer's assessment. For printing density, length, width, and height, Levels 1 and 3 were chosen as the extremes. The level 2 was determined by the DOE Minitab 22 tool to maximise the information obtained per experiment. In the case of the geometric variables, the minimum and maximum variables were chosen because of their possible applications in parallel robots, as shown in Figures 5 and 6. The raster angle was chosen as 0 and 45 degrees, and 90 degrees was omitted, given that previous research [4] found it behaved similarly to the 0-degree condition. The three levels of the layer thickness correspond to Ultimaker's Cura 5 software, super, dynamic, and standard quality prints. The levels are shown in Table 4.

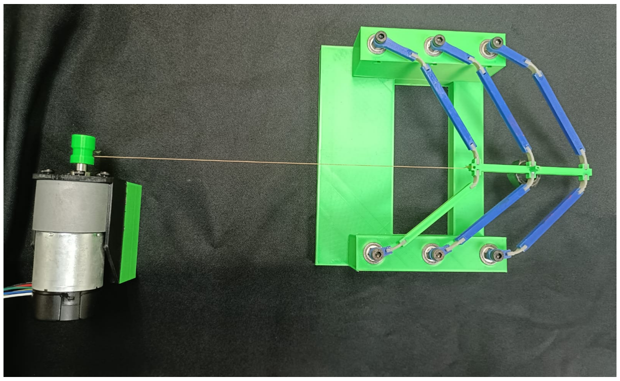

Figure 5: Flexible joint robot with multiple kinematic chains.

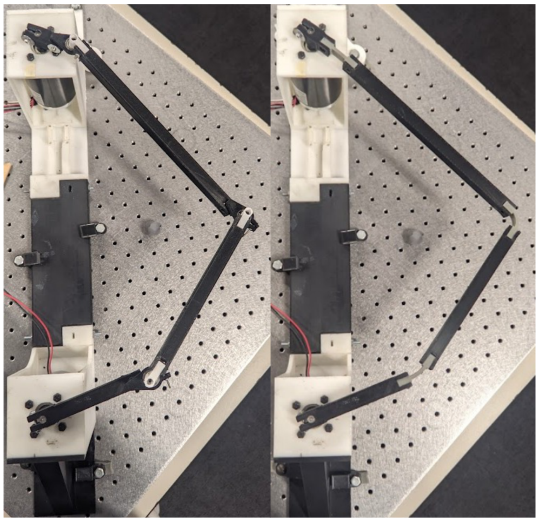

Figure 6: a) Bearing joint robot. b) Flexible joint robot.

Table 4: Parameters and their levels

Parameters	Levels		
	1	2	3
Printing density (%)	20	60	100
Length l (mm)	18	24	30
Width b (mm)	2	4.5	7
Height h (mm)	4	9	14
Layer thickness (mm)	0.12	0.16	0.2
Raster angle (°)	0	45	–

The roll of material was shown in Figure 1(a) mounted onto the 3D printer.

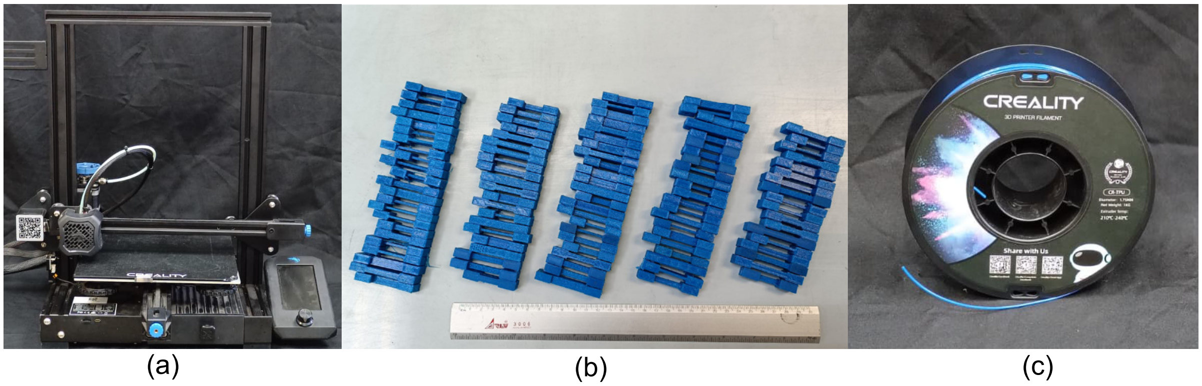

Figure 7: **Printer and printed parts.** (a). Creality Ender 3 V2 3D printer. (b). 3D-printed TPU test specimens. (c). Material used for the 3D-printed specimens

Author action:

In section 2.2 Design of experiments, a new 4th paragraph was added to describe the parameter selection process. Figure 1(a) was changed, and a Figure 1(c) was added to show the material used. The description of Figure 1 was updated to include the 1(c)image description as follows: "**Printer and printed parts.** (a). Creality Ender 3 V2 3D printer. (b). 3D-printed TPU test specimens. (c). Material used for the 3D-printed specimens". The second paragraph in section 2.1, Materials and methods, was updated to include the reference to the updated image 1: "Fig 1 presents the printer, printed specimens with the different levels of their parameters, and the material used.".

1.24 Reviewer # 3, Concern # 4

In the Design of Experiment section:

- **The reason for selecting certain parameters, such as frequency range, is not clearly justified. The rationale behind these choices should be explicitly stated.**
- **The mathematical explanations (spring-damper model) are not well-integrated with the practical aspects (arm motion, hub, experiments). It would be more appropriate to present the modelling section separately.**
- **The application of the equations is not clearly described. It must be explicitly stated how the damping and stiffness coefficients are obtained from the experiment.**

Author response:

Many thanks to the reviewer for highlighting this point. Standard DC motors do not respond equally to all input frequencies. They follow a low-pass behavior, responding to low-frequency inputs but not to high-frequency ones due to inertia and electrical inductance. The used motor has a maximum response frequency of 30 Hz, but 2 Hz was considered fast enough for a pick-and-place application based on trajectory planning optimization [9] were a pick-and-place cycle takes 2s.

The proposed model for the flexible joint is given by Eqs (4) and (5):

$$\mathbf{M}\ddot{r}(t) + \mathbf{C_F}\dot{r}(t) + \mathbf{K_F}r(t) = F(t) \tag{4}$$

$$\mathbf{I}\dot{\omega}(t) + \mathbf{C}_\tau\omega(t) + \mathbf{K}_\tau\Theta(t) = \tau(t) \tag{5}$$

The values in the matrices $\mathbf{M}$, $\mathbf{C_F}$, $\mathbf{K_F}$, $\mathbf{I}$, $\mathbf{C}_\tau$, $\mathbf{K}_\tau$, are calculated using the minimum squared formulae.

$$\hat{\beta} = \left(\mathbf{X^T X}\right)^{-1}\mathbf{X^T y} \tag{6}$$

where $\mathbf{X}$ is the input variables, $\mathbf{y}$ are the output variables and $\hat{\beta}$ are the estimated coefficients $\mathbf{M}$, $\mathbf{C_F}$, $\mathbf{K_F}$, $\mathbf{I}$, $\mathbf{C}_\tau$, $\mathbf{K}_\tau$. In the case of the proposed experiment, the inputs are the positions r, $\dot{r}$, $\ddot{r}$, Θ, ω, $\dot{\omega}$, and the outputs are the forces and torques F, τ. All the values used are measured according to the design of the experiment proposed in Section 1.2. Meaning the lattice structures are not modelled, and the flexible joint is considered homogeneous.

A video example of the experiment is shown in the following video `https://youtu.be/PuYnzHvUt_I`.

Author action:

- In section 2.2, paragraph three, an explanation about the frequency range was added, the explanation reads as follows: "The hub moves in a sinusoidal pattern with an amplitude of 1 rad, generating a load that is applied periodically at an increasing frequency rate starting from 0.2 Hz up to 1.85 Hz at the end of the experiment. The frequency rate is picked according to similar research in trajectory planning for robots [46]. A complete run of the experiment lasts for 2200 seconds.".

- The proposed model was removed from the design of experiment section, and a new section 1 "Proposed mass-spring-damper model" was added, The new section 1 includes the model assumptions, and the least squares problem inputs and outputs.

- The model description was expanded to describe how the coefficients are obtained explicitly as follows "The most commonly used model for flexible joints in mechanisms is the pseudo-rigid-body model [42]. The pseudo-rigid-body model approximates the behaviour of the flexible joints as a spring. The approximation is limited to a region of operation. Assuming the internal lattice structures are homogeneous, the behaviour of the flexible joint can be approximated by the proposed mass-spring-damper model.".

1.25 Reviewer # 3, Concern # 5

In the Results and discussion:

- **The section referring to outlier removal should clearly explain the method used to identify these data points. For instance, it should specify whether outliers were detected based on certain statistical criteria, such as variance thresholds, interquartile range (IQR), or Z-score.**

- **Although the nonlinear behavior of the joints is mentioned, the proposed model is based on linear assumptions. While this may be valid for small deformations, the scientific justification for adopting a linear model should be clearly provided. For example, presenting a tensile test diagram of the samples and defining the linear operational range would help support the model's applicability.**

Author response:

We appreciate and agree with the reviewer's observations. The outliers were removed by eliminating datasets with zeroes, repeated data, and NaN values, and the outliers were deleted using DBScan. The parameters used for the DBScan are $\epsilon = 0.5$ and a minimum cluster of 5 points.

Regarding the nonlinear behavior of TPU based joints. It is true that thermoplastic polyurethane exhibits nonlinear stress-strain characteristics, especially at larger strain %. In our study the operational regime of the joints remains within the small deformation range, where the stress-strain response of TPU can be approximated as linear.

Similar studies [8] deal with the strain using similar materials, the resulting engineering stress-strain curve shown in figure 8, indicates that TPU exhibits an approximately linear elastic region up to $100 - 150\%$ strain. Beyond this range, nonlinear stiffening becomes dominant, as expected for elastomeric materials. Since the maximum strains observed in our system remain well within this linear range, a linear constitutive model is appropriate for the intended operational conditions.

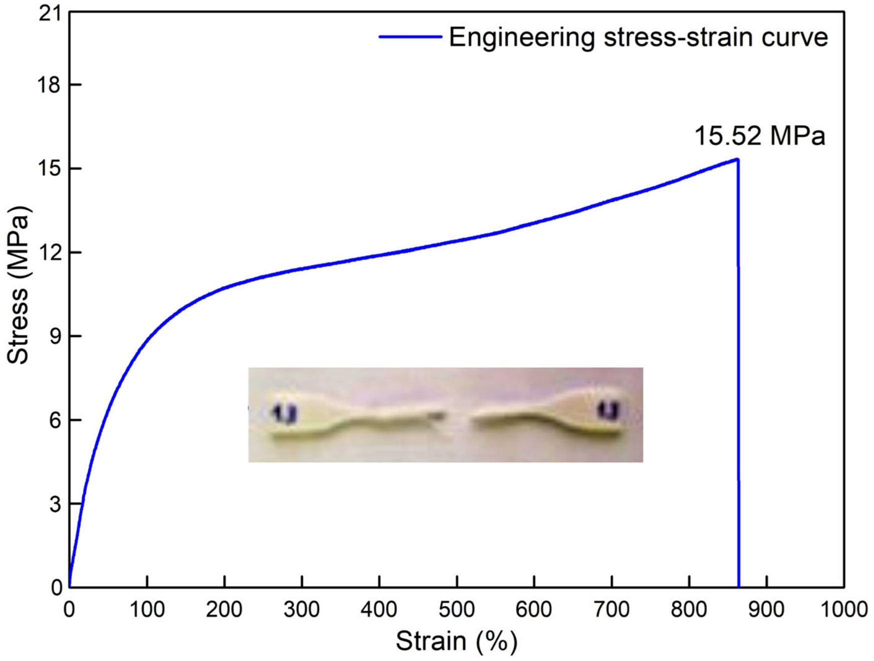

Figure 8: **Engineering stress-strain curves of sample 1.3 in group No.1 of article [8]**

In the case of this article the approximation is limited to the region of operation 1 rad of the sinusoidal amplitude, well within the limit of the pseudo-rigid-body model(PRBM) approximation as shown in [12] where the maximum angular deformation used is 75 deg. The spring coefficient depends on the maximum deformation of the compliant joint [11]. The obtained values for the proposed mass-spring-damper model

are only valid in this 1 rad region of deformation.

Author action:

In section 3 Results an discussion, the method for outlier removal was added and clearly stated in the first paragraph: "All the experiments were performed using the experimental Box-Behnken design. The results of the 160 test specimens were filtered and cleaned, eliminating zeroes, repeated data, and outliers, the outliers were deleted using DBScan. The parameters used for the DBScan are $\epsilon = 0.5$ and a minimum cluster of 5 points. The data cleaning results in 44 usable datasets.".

In the new section 1 "Proposed mass-spring-damper model" a description of the proposed model and assumptions made was done as follows: "The most commonly used model for flexible joints in mechanisms is the pseudo-rigid-body model [42]. The pseudo-rigid-body model approximates the behaviour of the flexible joints as a spring. The approximation is limited to a region of operation. Assuming the internal lattice structures are homogeneous, the behaviour of the flexible joint can be approximated by the proposed mass-spring-damper model.".

1.26 Reviewer # 3, Concern # 6

In the Conclusion:

- **Long and complex sentences should be rewritten into simpler ones with clearer structure to improve readability.**
- **The logical flow of information should be maintained (objective, method, results, interpretation) to enhance coherence.**
- **Statistical interpretations (e.g., p-values) should be presented more precisely and understandably, so that readers can clearly grasp the significance of the findings.**

Author response:

The insight offered by the reviewer has been helpful in rewriting the conclusions.

Author action:

- The conclusions were rewritten to avoid lengthy and complex sentences and improve readability.
- Values are removed from the conclusions to avoid repeating the results and discussion section and substituted with precise descriptions for example: "Increasing the printing density has the most

influence on the damping instead of the spring coefficients.", "The raster angle has the most influence on the spring coefficients, being the highest at 0°.", "A finer layer thickness increases both the damping and spring effect.".

- A new paragraph was added to the conclusions to present the P-values in a precise way: "The MANOVA showed the accuracy of the model and the importance of each parameter. The P-values were below 0.05 for the layer, width, and height. Among the examined parameters, the geometry parameters have the most significant roles in modifying the damping and spring coefficients, with the height being the most significant and the length the least important.".

References

[1] H. Sadaghian, S. Khalilzadehtabrizi, M. Farzam, and S. Dehghan, "Behavior of 3d-printed polymers under monotonic torsion–a database of 15 different materials," *Additive Manufacturing*, vol. 60, p. 103251, 2022.

[2] C. Zhang, T. Li, Q. Deng, and X. Li, "Compression behavior of 3d printed polymer tpu cubic lattice structure," *Materials Research*, vol. 25, p. e20220060, 2022.

[3] H. Kim and S. Lee, "Mechanical properties of 3d printed re-entrant pattern with various hardness types of tpu filament manufactured through fdm 3d printing," *Textile Science and Engineering*, vol. 57, no. 3, pp. 166–176, 2020.

[4] J. Kechagias and S. Zaoutsos, "Effects of 3d-printing processing parameters on fff parts porosity: Outlook and trends," *Materials and Manufacturing Processes*, vol. 39, no. 6, pp. 804–814, 2024.

[5] T. Xu, W. Shen, X. Lin, and Y. M. Xie, "Mechanical properties of additively manufactured thermoplastic polyurethane (tpu) material affected by various processing parameters," *Polymers*, vol. 12, no. 12, p. 3010, 2020.

[6] V. Marco, G. Massimo, and G. Manuela, "Additive manufacturing of flexible thermoplastic polyurethane (tpu): enhancing the material elongation through process optimisation," *Progress in Additive Manufacturing*, vol. 10, no. 4, pp. 2877–2891, 2025.

[7] B. Arifvianto, T. N. Iman, B. T. Prayoga, R. Dharmastiti, U. A. Salim, M. Mahardika, and Suyitno, “Tensile properties of the fff-processed thermoplastic polyurethane (tpu) elastomer,” *The International Journal of Advanced Manufacturing Technology*, vol. 117, no. 5, pp. 1709–1719, 2021.

[8] D. Le, C. H. Nguyen, T. H. N. Pham, V. T. Nguyen, S. M. Pham, M. T. Le, and T. T. Nguyen, “Optimizing 3d printing process parameters for the tensile strength of thermoplastic polyurethane plastic,” *Journal of Materials Engineering and Performance*, vol. 32, no. 23, pp. 10 805–10 816, 2023.

[9] F. Bourbonnais, P. Bigras, and I. A. Bonev, “Minimum-time trajectory planning and control of a pick-and-place five-bar parallel robot,” *IEEE/ASME Transactions on Mechatronics*, vol. 20, no. 2, pp. 740–749, 2014.

[10] J. Chimento, C. Lusk, and A. Alqasimi, “A 3-d pseudo-rigid body model for rectangular cantilever beams with an arbitrary force end-load,” in *International Design Engineering Technical Conferences and Computers and Information in Engineering Conference*, vol. 46360. American Society of Mechanical Engineers, 2014, p. V05AT08A029.

[11] S. Šalinić and M. Bižić, “A pseudo-rigid-body approach for dynamic analysis of planar compliant mechanisms,” in *Structures*, vol. 71. Elsevier, 2025, p. 108012.

[12] M. Mohammadi, A. Z. Kouzani, M. Bodaghi, and A. Zolfagharian, “3d-printed programmable bistable mechanisms for customized wearable devices in tremor attenuation,” *Journal of the Mechanical Behavior of Biomedical Materials*, p. 107006, 2025.
