# Supplementary material for: Flexible joints performance assessment of additive manufacturing FDM 3D printed TPU
Source: PLoS One. 2025 Nov 13;20(11):e0336401. doi: 10.1371/journal.pone.0336401 (PMC12614568; doi:10.1371/journal.pone.0336401)
Supplement: Attachment — Submitted filename: Response_to_reviewers_auresp_2.pdf [file pone.0336401.s003.pdf]

Original Manuscript ID: PONE-D-25-26891
Original Article Title: "Flexible joints performance assessment of additive manufacturing FDM 3D printed TPU."

To : Ph.D Himadri Majumder, *PLOS ONE Academic Editor*
Re : Response to reviewers

Dear Editor,

Thank you for allowing a resubmission of our manuscript, with an opportunity to address the reviewers' comments.
We are uploading (a) A rebuttal letter that responds to each point raised by the academic editor and reviewers, labeled 'Response to Reviewers'. (b) A marked-up copy of the manuscript that highlights changes made to the original version, labelled 'Revised Manuscript with Track Changes'. (c) An unmarked version of the revised paper without tracked changes labelled 'Manuscript'.

We trust that the revised manuscript now meets the reviewers' expectations. We again thank the reviewers for their valuable insights, which significantly improved the quality of this work.

Best Regards,

Daniel Rodríguez Flores, et al.

1 Response to reviewers:

The authors appreciate all reviewers' comments and suggestions and are confident that the corrections outlined in this document fulfill their expectations.

It is worth mentioning that cross-references to bibliography and figures refer to this document (Response to Reviewers) and only when specified, refer to those of the newly submitted manuscript.

1.1 Reviewer # 2: Concern # 1

A. In the revised manuscript, the following concern is not adequately explained by the authors. Please, Table 3 is Table 3, not Table 2. Explain how to implement BBD given a parameter with 2 levels. Also, explain better the infill type and why 0 and 90 degrees are the same.

Author response:

The traditional Box-Behnken (BBD) design of experiments (DOE) does not allow for the use of both continuous and categorical factors. For the continous factor the general surface response model is given by equation (1).

$$y = \beta_0 + \sum_{i=1}^{4} \beta_i x_i + \sum_{i=1}^{4} \beta_{ii} x_i^2 + \sum_{i<j}^{4} \beta_{ij} x_i x_j + \varepsilon \tag{1}$$

where y is the response variable, x_i are the continous factors, β_0 is the intercept term, β_i are the linear coefficients associated with each continuous factor, β_{ii} corresponds to the quadratic coefficients modeling curvature, β_{ij} are the interaction coefficients between continuous factors x_i and x_j, ε is the experimental error term.

Using the DOE minitab 22 tools of response surface design with the factors shown in Table 1, its possible to build a BBD design where the design is blocked for the categorical levels an for each of the categories combinations repeats or fractions the BBD, the result is what it seems to be a mixed BB when in reality are multiple partial BBD combined per categorical levels. Althoug Minitab does not publicly document each source algorithm, according to its DOE technical manual and its official training materials, the baseline references include [1, 2]. The mixed surface response design equation is given in (2).

$$y = \beta_0 + \sum_{i=1}^{4} \beta_i x_i + \sum_{i=1}^{4} \beta_{ii} x_i^2 + \sum_{i<j}^{4} \beta_{ij} x_i x_j + \sum_{k=1}^{K} \gamma_k Z_k + \sum_{i=1}^{4} \sum_{k=1}^{K} \delta_{ik} x_i Z_k + \varepsilon \tag{2}$$

where Z_k are the indicator variables for the categorical factors, δ_{ik} are the interaction coefficients between continuous factors x_i and categorical factors Z_k, γ_k are the coefficients associated with the principal effect of the categorical factors.

For categorical factors, they are treated as fixed effects (similar to ANOVA), without quadratic terms. A summary of the DOE is shown in Figure 1. The complete DOE Minitab 22 file can be found in: `https://simtk.org/projects/flex-tpu` in the documents section.

Table 1: Factors

Continous factors

**Factor**	**Name**	**Low**	**High**
**A**	Density	20	100
**B**	Length	18	30
**C**	Height	2	7
**D**	Width	2	7

Categorical factors

**Factor**	**Name**	**Levels**	**Values**		
**E**	Layer	3	0.12	0.16	0.2
**F**	Angle	2	45	0	

Design Summary

Factors:	6		
Base runs:	162	Replicates:	1
Base blocks:	1	Total blocks:	1
Central points:	18		

Figure 1: Design Summary.

The chosen infill type is cubic, where the 3D lattice pattern resembles cubes rotated in space. The main advantages include being strong and lighter in all directions compared to a grid with similar strength. Its use is most common in functional mechanical parts, especially under torsion [3].

Even though the 0° and 90° orientations look completely different, as shown in Figure 2, the 3D printer's two planar degrees of freedom allow it to print the specimen in the same manner. Instead of the extruder moving along the hotbed, the hotbed moves along the extruder. A video showing the extruder path during the printing process is shown in the following link: `https://youtu.be/3exTFhJNu4M`.

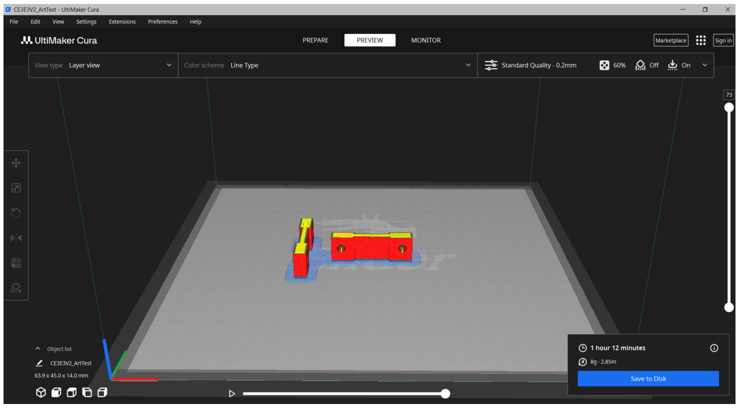

Figure 2: Printing direction.

Author action:

In section 2.2, paragraphs 4 and 5, the description of the DOE tool used was updated, the new description includes the mention of the categorical factors.

1.2 Reviewer # 2, Concern # 2

- **Table 3. Please give the details of the experimental design. Factors, center point, etc. Give a reference that uses the same BBD experimental array, or explain where to find or how construct this experimental array.**
- **Please explain the following sentence. 'The results of the 160 test specimens were filtered and cleaned, eliminating zeroes, repeated data, and outliers, resulting in only 44 usable datasets.'**

Author response:

- The factors are density, length, height, width, layer thickness, raster angle, the last two being categorical factors with two and three levels, respectively.

The complete list of centerpoints is shown in Table 2, and can also be found in: `https://simtk.org/projects/flex-tpu`. In the DEO Minitab file, the centerpoints are marked in column 3 by a zero. Given the DOE methodology described in concern 1, the resulting four continuous factors and two categorical factors with two and three levels means there are six combinations, times three centerpoints, resulting in 18 total centerpoints, meaning six unique combinations each repeated 3 times.

Table 2: Centerpoints

Run	Density	Length	Height	Width	Layer	Angle
25	60	24	4.5	4.5	0.12	45
26	60	24	4.5	4.5	0.12	45
27	60	24	4.5	4.5	0.12	45
52	60	24	4.5	4.5	0.16	45
53	60	24	4.5	4.5	0.16	45
54	60	24	4.5	4.5	0.16	45
79	60	24	4.5	4.5	0.2	45
80	60	24	4.5	4.5	0.2	45
81	60	24	4.5	4.5	0.2	45
106	60	24	4.5	4.5	0.12	0
107	60	24	4.5	4.5	0.12	0
108	60	24	4.5	4.5	0.12	0
133	60	24	4.5	4.5	0.16	0
134	60	24	4.5	4.5	0.16	0
135	60	24	4.5	4.5	0.16	0
160	60	24	4.5	4.5	0.2	0
161	60	24	4.5	4.5	0.2	0
162	60	24	4.5	4.5	0.2	0

- The original design of experiments results in 162 datasets. To ensure the validity of the datasets, some reading errors result in full files or columns of zeroes or NaN values, making them useless for the analysis. In the case of repeated data, the wall thickness is 1 mm, so when the variable width b is 2 mm, the printing density becomes 100%, resulting in an overlap of the data. The outliers were

detected using DBScan. The parameters used for the DBScan are $\epsilon = 0.5$ and a minimum cluster of 5 points. Once the discrimination process is completed, the results show 44 usable datasets shown in Table 3: Experimental data.

Author action:

In Section 3: Results and discussion, paragraph 1, the description of the data filtering was updated.

References

[1] R. H. Myers, D. C. Montgomery, and C. M. Anderson-Cook, *Response surface methodology: process and product optimization using designed experiments.* John Wiley & Sons, 2016.

[2] D. C. Montgomery, *Design and analysis of experiments.* John wiley & sons, 2017.

[3] P. K. Mishra, P. Senthil, S. Adarsh, and M. Anoop, "An investigation to study the combined effect of different infill pattern and infill density on the impact strength of 3d printed polylactic acid parts," *Composites Communications*, vol. 24, p. 100605, 2021.
