# Supplementary material for: Flexible joints performance assessment of additive manufacturing FDM 3D printed TPU
Source: PLoS One. 2025 Nov 13;20(11):e0336401. doi: 10.1371/journal.pone.0336401 (PMC12614568; doi:10.1371/journal.pone.0336401)
Supplement: Attachment — Submitted filename: Comments.docx [file pone.0336401.s001.docx]

Comments:

**Abstract Clarity**:
The abstract provides a general overview but lacks clarity in explaining the novelty of the work. Consider explicitly stating what makes this study unique compared to existing literature on flexible joints or lattice-based sandwich structures.

**Introduction Depth**:
The introduction mentions several prior works but could benefit from a more detailed gap analysis. Please elaborate on what specific problem remains unsolved and how this study addresses that gap.

**Figure Quality and Captions**:
Some figures (e.g., Figures 2, 4, and 8) have relatively low resolution and unclear captions. Improve image quality and ensure all abbreviations or variables are clearly defined in the captions.

**Methodological Details**:
The authors should provide more detailed information about the material properties used in the simulations, including elastic modulus, Poisson’s ratio, and any assumptions made during modeling. Were the lattice structures modeled as homogeneous materials?

**Validation of FE Model**:
While the paper includes numerical results, experimental validation of the finite element (FE) models is lacking. Even a brief comparison to literature data or a previous study would strengthen the reliability of the simulations.

**Discussion on Limitations**:
The paper would benefit from a section discussing the limitations of the current approach—both in terms of simulation assumptions and possible real-world manufacturing constraints.

**Clarity in Results Section**:
The results section discusses stress distribution and stiffness but should also discuss possible implications on design, such as trade-offs between flexibility and strength, or how joint orientation might impact fatigue life.

**Grammar and Language**:
The manuscript requires minor grammatical corrections for fluency and readability. For example, replace “this paper investigates the joint flexibility…” with “This paper investigates the flexibility of joints…”

**Conclusions Enhancement**:
The conclusions currently restate results without highlighting the significance. Consider briefly noting how these findings can influence future additive manufacturing design of lattice-based sandwich structures.

**References**:
Ensure that all references are formatted consistently and up to the journal’s guidelines. Double-check citations for completeness
